# Tripterygium and its plant extraction for systemic lupus erythematosus: A protocol for systematic review and meta analysis

**DOI:** 10.1097/MD.0000000000021909

**Published:** 2020-08-21

**Authors:** Fangying Chen, Junting Liu, Zhimin Zhao, Ziping Li, Kuanyu Wu

**Affiliations:** a Continuing Education Division, the First Affiliated Hospital of Guangzhou University of Chinese Medicine; b The First Clinical College, Guangzhou University of Chinese Medicine; c Acupuncture and Moxibustion Department, Guangdong Provincial Hospital of Chinese Medicine, Guangzhou, Guangdong Prov.; d Rheumatism Department, the Second People's Hospital of Fujian Province, Fujian Prov., China.

**Keywords:** protocol, systematic review, systemic lupus erythematosus, tripterygium

## Abstract

**Background:**

Systemic Lupus Erythematosus (SLE) is a diffuse connetive tissue disease, which is difficult to be conquered. However, the traditional Chinese medicine is significant in the treatment. And the Chinese medicine tripterygium and its plant extraction can help us to overcome this disease, to some extent.

**Methods:**

The deadline should be from inception to February 2020 by computer from the databases: the Cochrane Library, Pubmed, Embase, Web of Science in English and the Chinese National Knowledge Infrastructure, Wanfang Database, Chinese Biomedical Literature Database and Chinese Science, Chinese Traditional Medicine Database, Chinese Science and Technology Periodical Database in Chinese. Included criteria are randomized controlled trials. The primary outcomes are the clinical symptoms, systemic lupus erythematosus disease activity index and quality of life questionnaire (the top 10 frequency). We will use RevMan 5.0 statistical software for data synthesis, sensitivity analysis, meta regression, subgroup analysis, and risk of bias assessment. The publish bias will be assessed by a funnel plot and the funnel plot symmetries will be evaluated by Begg and Egger tests. We will use the Grading of Recommendations Assessment, Development and Evaluation system to assess the quality of evidence.

**Results:**

This article will give a protocol for meta analysis which can make sure the efficacy and side effect of the tripterygium and its plant extraction for SLE.

**Conclusion:**

The efficacy and side effect of the tripterygium and its plant extraction for SLE will be evaluated.

**Ethics and dissemination:**

Without personal information involved, ethical approval and informed consent form is no need. The review will be submitted to a peer-reviewed journal prospectively to spread our findings.

**PROSPERO registration number:**

PROSPERO CRD42020176444

## 1 Introduction

Systemic lupus erythematosus (SLE) is, to some extent, a kind of diffuse connective tissue disease, which can be involved in all systems of the whole body. Its heterogeneity make heritage scientists, rheumatoid expert, clinicians…be in a dilemma.^[1]^ For its heterogeneity, the treatment of disease is still a worldwide problem.^[2]^ Western medicine treatment mainly includes immunosuppression and immunomodulation. Excessive reliance on corticosteroids remains the main cause of high failure rates.^[3]^ With the health need growing by leaps and bounds, people find that traditional Chinese medicine can not only help us ameliorate the symptoms, but also a strategy for a holistic approach to life in SLE.

Tripterygium wilfordii as an extractive of the traditional Chinese medicine can completely inhibit tumor necrosis factor α induced oxidative rupture and NET formation of neutrophils. Tripterygium wilfordii also completely inhibited purified immunoglobulin G induced oxidative rupture and NET formation of neutrophils in serum of patients with SLE. All suggest that tripterygium wilfordii may have the potential function to treat neutrophil and NETs inflammatory and autoimmune diseases.^[4]^ Another report reveal that the tripterygium can significantly stimulate NK cell killing activity, which is dose dependence.^[5]^

This paper considers the randomized controlled trials (RCTs) literature as the research object, adopts the evaluation, and stipulates that more than 3 points are high quality documents. When it come across the outcomes, the studies choose the highest 10 frequency indicators. Elaborate the results of the study, the author find it mainly focused on the indicators related to the SLE disease activity index (SLEDAI) score,^[6]^ including the observation of the clinical manifestations of joint symptoms, lupus nephritis, lupus brain related diseases, and the study of the laboratory indicators of lupus nephritis, thrombocytopenia, erythropenia, aleucocytosis and so on.^[7,8]^ Furthermore, there are still relevant investigations of cytokines that can not be ignored, such as interleukin 18,^[9,10]^ and complement C3, C4.^[11]^ The application of the scale is typically the Quality of Life Questionnaire (QoL questionnaire), which covers the study of biological, psychological and social medical models.^[12]^ Some articles have even approach to the toxic side effects of Tripterygium, suggesting that it causes bone mineral density decline.^[13]^ To conclude above, the author find that Tripterygium as a representative drug for the treatment of SLE, its efficacy and side effects are worth to deepen research.

## 2 Method

### 2.1 Eligibility criteria

#### 2.1.1 Article type

(1) RCTs;
(2) with at least a comparable group;
(3) Editorials, reviews, animal experiments, observational cohort will be withdrawn;
(4) Deadline: From inception to February 2020;
(5) Language: English and Chinese.

#### 2.1.2 Participants type

(1) the ones who are diagnosed with SLE;
(2) They are above 18 years old to eliminate the “Lupus Erythematosus in Children”;
(3) Initially, the patients are with a scores equal or above 5 in SLEDAI to make sure the patients should be intervened;

#### 2.1.3 Interventions

The intervention measures in the treatment group are traditional Chinese medicine capsule, decoction, pill, traditional Chinese medicine injection and they are combined or not combined with western medicine treatment. The control group take western medicine alone or placebo.

#### 2.1.4 Outcomes

The top ten frequency results in documents will be applied, such as, the SLEDAI, the QoL questionnaire …the primary outcomes are the clinical symptoms, SLEDAI and QoL questionnaire,…up to 10. The secondary outcomes are the loss ratio of follow-up, the adverse reactions.

### 2.2 Search methods for the identification of studies

Once the information is not complete, we will give the author a call, an email or even pay a visit to them to accomplish the material of RCTs. Totally 2 researches (FY and JT) are searching the documents and collecting the material in the database, separately. If there are in a dilemma, they will hand up the material to ZM who revise and decide how to deal with them.

Database: The Cochrane Library, Pubmed, Embase, Web of Science in English and the Chinese National Knowledge Infrastructure, Wanfang Database, Chinese Biomedical Literature Database and Chinese Science, Chinese Traditional Medicine Database, Chinese Science and Technology Periodical Database in Chinese.

Entry Terms: Lupus Erythematosus, Systemic, SLE, Lupus Erythematosus Disseminatus, Libman-Sacks Disease, Disease, Libman-Sacks, Libman Sacks Disease, Tripterygium, Tripterygiums, Tripterygium hypoglaucum, Tripterygium hypoglaucums, hypoglaucums, Tripterygium, Tripterygium wilfordii, Tripterygium wilfordius, wilfordius, Tripterygium, Leigong Teng, Leigong Tengs, Teng, Leigong, Tengs, Leigong, Thundergod Vine, Thundergod Vines, Vine, Thundergod, Vines, Thundergod, Kunmingshanhaitang, Huobahuagen, Nansheteng, Nanshegen, Duanchangcao, Huangtenggen, Huangyao, Shuimangcao, Caichongyao, Sanlenghua.

Search use keywords for the Cochrane Library, Embase, Web of Science and the Chinese National Knowledge Infrastructure, Wanfang Database, Chinese Biomedical Literature Database and Chinese Science, Chinee Science and Technology Periodical Database and combine the mesh terms with the “All Fields” in Pubmed and Chinese Traditional Medicine Database.

Additional material can not be neglected. They include in Conference proceeding, Hand searching, those owing to Chinese Clinical Trial Registry or International Clinical Trial Registry Platform.

### 2.3 Data processing

#### 2.3.1 Selecting studies

**Figure 1:**
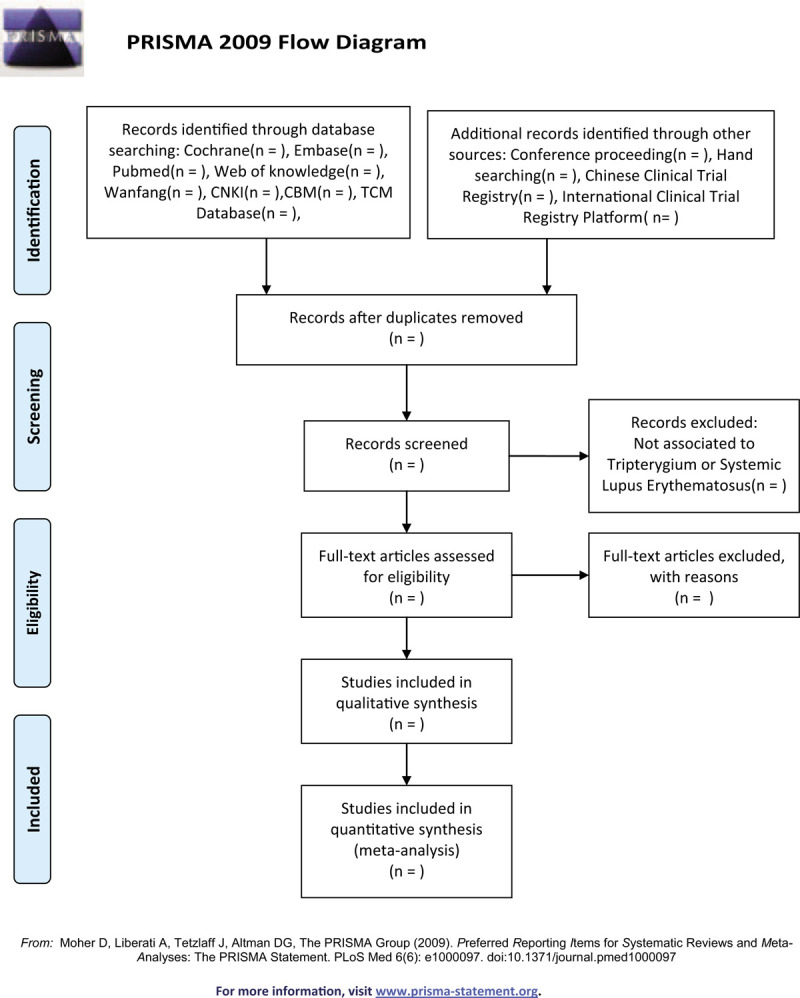
PRISMA flow diagram to describe the search process. PRISMA= Preferred Reporting Items for Systematic Review and Meta-Analyses.

Once the article from either electronic resources or supplementary material can not be complete even after a connection with the authors, it will be deleted in our research. Additionally, we will move away the duplicates and the ones which are not connected with our research. The ones will be removed if they are not accordance with the conditions we have proposed above (Fig. 1).

#### 2.3.2 Data extraction and management

Two authors (FY and JT) go through the data extraction using a questionnaire which is proposed by the correspondence author (KY). The characters which will be selected are: name of the first author, publication year, the scores of Jadad evaluation, type of studies, number of groups, intervention measures, course, outcomes and adverse events.

#### 2.3.3 Data analyse

The RevMan 5.0 statistical software provided by Cochrane the International Network of Evidence-based Medicine will be used for meta analysis. Odds ratios or risk ratios is the outcome analysis statistic for categorical data. While weighted mean difference or standardized mean difference will be adopted in continuous data. And the interval estimates are expressed using 95% confidence interval. The heterogeneity among the included studies will be tested by *χ*^2^. If there is statistical homogeneity (*P* > .1, *I*_2_<50%) among the studies, the fixed effect model will be used to analyze; if there is heterogeneity, the causes of the heterogeneity will be analyzed, and the factors that might lead to heterogeneity will be analyzed by setting up subgroups. If there is statistical heterogeneity among the included studies, but no clinical heterogeneity or difference have no clinical significance (*P* < .1, *I*_2_ > 50%), a random effect model will be used for analysis. If the heterogeneity between groups is too large, descriptive analysis will be used. Analyzing of publication bias use funnel plots. Heterogeneity, which is generated from low quality studies, can process sensitivity analysis test to exam the stability of the results.

#### 2.3.4 To assess the bias risk in included studies

The bias risk has been divided into “low”, “high,” “unclear (unknown or unclear)”. We use the Cochrane Handbook for Systematic Review of Interventions V.5.1.0 to assess the bias risk in included studies. For these aspects should be considered when come across this evaluation for the quality assessment: the random sequence generation, allocation concealment, blinding of outcomes, blinding of participants, therapists and assessors; incomplete data; and selective outcome reporting; other sources bias.

#### 2.3.5 To evaluate the publication bias

A funnel plot will be adopted to evaluate the report bias related to SLE. Begg and Egger tests can estimate the symmetry of funnel plot.

#### 2.3.6 Quality of evidence

The Grading of Recommendations Assessment, Development, and Evaluation will be used to evaluate the quality of evidence (high,moderate,low, very low). The quality will be reduced by a low exactness, poor design with a lot of limitations, inconsistencies, deviation, unexplained heterogeneity or publication bias.

#### 2.3.7 Patient or public involvement

Non.

## 3 Discussion

SLE is a systemic multiple tissue and organs involved disease that can endanger life for patients. The latest treatment for SLE is genotype targeted therapy, but clinical evidence is insufficient and its cost is expensive.^[14,15]^ The most widespread anti-rheumatic drugs are traditional disease modifying antirheumatic drugs and bio- disease modifying antirheumatic drugs, which increase the risk of tumorigenesis and infection.^[16–18]^ And the status of Chinese medicine in the treatment of SLE can not be underestimated. Traditional Chinese medicine almost uses tripterygium, caulis sinomenii and futokadsura stem (pepper stem). And with the highest efficacious against SLE is Tripterygium wilfordii.^[19–21]^ Although it has a reproductive system toxicity, even some studies have pointed out that it can reduce bone density and may cause osteoporosis,^[13]^ it is still favored in clinical practice. For the most part of the studies on Tripterygium wilfordii or Tripterygium wilfordii extracts, or Tripterygium wilfordii polyglycoside tablets, are conducted in China. The studies are compared with traditional anti-rheumatic drugs such as cyclophosphamide, Mycophenolate mofetil …or with traditional Chinese medicine….^[5,13]^ But the study compared with traditional Chinese medicine or other complimentary therapy will be eliminated in the meta analysis of this paper. A majority of the conclusions of these studies show the tripterygium to be effective.^[19,20]^ Meta analysis of the efficacy and side effects of Tripterygium wilfordii in SLE in Science Citation Index magazine has not been reported before. So it is worth further in-depth discussion.

## Author contributions

**Methodology:** Fangying Chen, Kuanyu Wu

**Supervision:** Ziping Li

**Writing – original draft:** Fangying Chen, Junting Liu, Zhimin Zhao

**Writing – review & editing:** Fangying Chen, Kuanyu Wu

## References

[1] Dorner T Furie R Novel paradigms in systemic lupus erythematosus. Lancet 2019;393:2344–58. 31180031 10.1016/S0140-6736(19)30546-X

[2] Morand EF Furie R Tanaka Y Trial of anifrolumab in active systemic lupus erythematosus. N Engl J Med 2020;382:211–21. 31851795 10.1056/NEJMoa1912196

[3] Durcan L O’Dwyer T Petri M Management strategies and future directions for systemic lupus erythematosus in adults. Lancet 2019;393:2332–43. 31180030 10.1016/S0140-6736(19)30237-5

[4] Yu Y Koehn CD Yue Y Celastrol inhibits inflammatory stimuli-induced neutrophil extracellular trap formation. Curr Mol Med 2015;15:401–10. 25941817 10.2174/1566524015666150505160743 PMC4527119

[5] Zhao XZ Effects of Astragalus membranaceus and Tripterygium hypoglancum on natural killer cell activity of peripheral blood mononuclear in systemic lupus erythematosus. Zhongguo Zhong Xi Yi Jie He Za Zhi 1992;12:669–71. 1301849

[6] Qiu YL HU L Wen G The impact factors analysis for Tripterygium wilfordii polyglycoside on ovary in lupus erythematosus patients. Guangdong Medical Journal 2011;32:3214–5.

[7] Weng MW Qiu BS Kang KF An analysis of 24 patients with IgA deposition at the BMZ. J Dermatol 1993;20:276–8. 8340531

[8] Shen K Yu Y Tang Z The prognosis of biopsy-proven lupus nephritis in chinese patients: long term follow-up of 86 cases. Chin Med J (Engl) 1997;110:502–7. 9594205

[9] Jin CY Hu GH Zheng BZ Sdudy on effect of tripterygium on plasma IL-18 content in patients with lupus nephritis. Zhongguo Zhong Yao Za Zhi 2008;33:1075–7. 18652362

[10] Hu N Lv XY Rao ZJ The effect of triptolide on expression of IL-2, IL-4, IFN-(in MRL/lpr Mice. China Foreign Medical Treatment 2010;29:5–6.

[11] Wu JH Zhu LF Qin WZ Effect of Tripterygium wilfordii on complement level of systemic lupus erythematosus. Acta Academiae Medicinae Shanghai 1996;23:472–3.

[12] Wang YY He HQ Clinical study of methylprednisolone pulse combined with Tripterygium Wilfordii Tablet in the treatment of systemic lupus erythematosus in children. J HBUM 2019;38:226–30.

[13] Huang L Feng S Wang H Decreased bone mineral density in female patients with systemic lupus erythematosus after long-term administration of Tripterygium Wilfordii Hook. F. Chin Med J (Engl) 2000;113:159–61. 11775543

[14] Yuan K Huang G Sang X Baricitinib for systemic lupus erythematosus. Lancet 2019;393:402. 10.1016/S0140-6736(18)32763-6 30712894

[15] Wallace DJ Furie RA Tanaka Y Baricitinib for systemic lupus erythematosus: a double-blind, randomised, placebo-controlled, phase 2 trial. Lancet 2018;392:222–31. 30043749 10.1016/S0140-6736(18)31363-1

[16] Lai GG Koo YX Tao M Use of rituximab in combination with high-dose methotrexate in the treatment of primary central nervous system lymphoma in a mycophenolate mofetil treated patient with lupus nephritis. Acta Oncol 2011;50:144–5. 20670084 10.3109/0284186X.2010.504231

[17] Md YM Vital EM McElvenny DM Predicting severe infection and effects of hypogammaglobulinemia during therapy with rituximab in rheumatic and musculoskeletal diseases. Arthritis Rheumatol 2019;71:1812–23. 31131994 10.1002/art.40937

[18] Ognenovski VM Marder W Somers EC Increased incidence of cervical intraepithelial neoplasia in women with systemic lupus erythematosus treated with intravenous cyclophosphamide. J Rheumatol 2004;31:1763–7. 15338497

[19] Kao NL Richmond GW Moy JN Resolution of severe lupus nephritis associated with Tripterygium wilfordii hook F ingestion. Arthritis Rheum 1993;36:1751–2. 8250996 10.1002/art.1780361217

[20] Zhang N Qin W Xu D Diagnosis and treatment of systemic lupus erythematosus with integrated traditional Chinese and Western medicine. Zhongguo Zhong Xi Yi Jie He Za Zhi 2000;20:883–7. 11938855

[21] Wang Y Yu C Zhang H Lipopolysaccharides-mediated injury to chondrogenic ATDC5 cells can be relieved by Sinomenine via downregulating microRNA-192. Phytother Res 2019;33:1827–36. 31094031 10.1002/ptr.6372

